# Follow up: Compound data sets and software tools for chemoinformatics and medicinal chemistry applications: update and data transfer

**DOI:** 10.12688/f1000research.3713.1

**Published:** 2014-03-11

**Authors:** Ye Hu, Jürgen Bajorath

**Affiliations:** 1 Department of Life Science Informatics, B-IT, LIMES Program Unit Chemical Biology and Medicinal Chemistry, Rheinische Friedrich-Wilhelms University, Bonn, D-53113, Germany

## Abstract

In 2012, we reported 30 compound data sets and/or programs developed in our laboratory in a data article and made them freely available to the scientific community to support chemoinformatics and computational medicinal chemistry applications. These data sets and computational tools were provided for download from our website. Since publication of this data article, we have generated 13 new data sets with which we further extend our collection of publicly available data and tools. Due to changes in web servers and website architectures, data accessibility has recently been limited at times. Therefore, we have also transferred our data sets and tools to a public repository to ensure full and stable accessibility. To aid in data selection, we have classified the data sets according to scientific subject areas. Herein, we describe new data sets, introduce the data organization scheme, summarize the database content and provide detailed access information in ZENODO (doi: 10.5281/zenodo.8451 and doi:10.5281/zenodo.8455).

## Introduction

The compound data sets reported in our original article ^1^ and the new data sets presented herein have resulted from research in the chemoinformatics and medicinal chemistry area and have mostly been generated from public domain repositories of compound structures and activity data. In addition, software tools made publicly available have also been developed in our laboratory ^1^. Data sets reported in the scientific literature in the context of computational method development and evaluation are often not publicly available, which limits the reproducibility of computational investigations and comparisons of different computational methods. We believe that it is important to provide such data to the scientific community to further improve the transparency and credibility of computational studies and support method development. In addition to the data sets designed for the development and evaluation of computational methods, we also make available data sets that were generated as a resource and knowledge base for medicinal chemistry applications. Our data sets and tools are provided via the ZENODO platform ( https://zenodo.org/) to ensure easy and stable access.

## Materials and methods

The data sets reported herein were predominantly generated from ChEMBL ^2, 3^, BindingDB ^4^ and PubChem ^5^ (a few exceptions are specified in the original data article ^1^). Compound structures are represented as SMILES ^6^ strings or SD files ^7^. Activity information and other (data set-dependent) annotations are provided in the individual data files. For software tools (written in different languages), the source code is also made available.

## Data description

Table 1 provides the updated list and classification of all freely available data sets and programs. Entries were organized according to the following scientific subject areas: data sets for structure-activity relationship (SAR) and structure-selectivity relationship (SSR) analysis, SAR visualization (SAR_VZ), and virtual screening via similarity searching or machine learning (VS_ML). In addition, the programs are provided separately (PROG). Data sets and programs are contained in separate ZENODO deposition sets with a unique reference. Three matched molecular pair (MMP)-based data sets also included in our update have recently been reported and described in detail ^8^. Entries 1–30 in Table 1 represent the data sets and programs that we initially provided *via* our website ^1^ and entries 31–43 represent new data sets. In the following, the new data sets are described:

**Table 1.** Data sets and programs.

Entry	Year	Subject area index label	Description
1 ^[9]^	2007	VS_ML_1	9 activity classes (AC) with increasing structural diversity
2 ^[9]^	2007	VS_ML_2	~1.44 million ZINC compounds used for various virtual screening trials
3 ^[10]^	2007	PROG_1	Molecular similarity histogram filtering
4 ^[11]^	2007	SSR_1	4 SD files with 26 selectivity sets; compounds are annotated with selectivity values for different targets
5 ^[12]^	2008	SSR_2	7 compound selectivity sets containing 267 biogenic amine GPCR antagonists
6 ^[13]^	2008	SSR_3	18 selectivity sets for targets from 4 families
7 ^[14]^	2008	VS_ML_3	25 sets of compounds of increasing complexity and size
8 ^[15]^	2009	VS_ML_4	242 hERG inhibitors
9 ^[16]^	2009	SSR_4	243 ionotropic glutamate ion channel antagonists
10 ^[17]^	2009	PROG_2	Combinatorial analog graph (CAG) program with a sample set consisting of 51 thrombin inhibitors
11 ^[18]^	2009	VS_ML_5	20 AC from the literature and 15 AC from the Molecular Drug Data Report
12 ^[19]^	2010	VS_ML_6	8 AC
13 ^[20]^	2010	PROG_3	Program to generate target selectivity patterns of scaffolds
14 ^[21]^	2010	PROG_4	Multi-target CAGs (see also entry 10) with a sample set containing 33 kinase inhibitors
15 ^[22]^	2010	PROG_5	SARANEA
16 ^[23]^	2010	PROG_6	3D activity landscape program with a sample set containing 248 cathepsin S inhibitors
17 ^[24]^	2010	SAR_1	2 sets of MMPs from BindingDB and ChEMBL
18 ^[25]^	2010	PROG_7	Similarity-potency tree (SPT) program with a sample set containing 874 factor Xa inhibitors
19 ^[26]^	2010	VS_ML_7	17 target-directed compound sets; each set contains a minimum of 10 distinct scaffolds and each scaffold represents 5 compounds
20 ^[27]^	2011	SAR_VZ	10,489 malaria screening hits
21 ^[28]^	2011	SAR_2	458 target-based sets with scaffolds and scaffold hierarchies
22 ^[29]^	2011	SAR_VZ	4 sets of compounds active against 3 or 4 targets
23 ^[30]^	2011	SAR_VZ	881 factor Xa inhibitors
24 ^[31]^	2011	VS_ML_8	50 AC prioritized for similarity searching
25 ^[32]^	2011	VS_ML_9	25 data sets from successful ligand-based virtual screening applications
26 ^[33]^	2011	SAR_3	26 conserved scaffolds in activity profile sequences of length 4
27 ^[34]^	2011	PROG_8	Scaffold distance function
28 ^[35]^	2011	SAR_4	2 sets of compounds with multiple K _i_ or IC _50_ measurements against the same targets that differed within 1 order of magnitude
29 ^[36]^	2012	SAR_VZ	4 AC
30 ^[37]^	2012	SAR_5	5 sets of different types of activity cliffs
31 ^[38]^	2012	VS_ML_10	50 AC for scaffold hopping analysis
32 ^[39]^	2012	SAR_6	61 AC consisting of SAR transfer series with regular potency progression
33 ^[40]^	2013	SAR_7	4 activity measurement type-dependent sets of scaffolds
34 ^[41]^	2013	VS_ML_11	2 multi-target compound sets
35 ^[42]^	2013	VS_ML_12	4 multi-target compound sets and 3 multi-mechanism sets
36 ^[43]^	2013	SAR_8	2337 compound series matrices
37 ^[44]^	2013	SAR_9	128 AC containing ≥100 compounds with K _i_ values
38 ^[45]^	2014	SAR_10	30,452 and 45,607 target-based MMS with K _i_ and IC _50_ values, respectively
39 ^[46]^	2014	SAR_11	221 drug-unique scaffolds
40 ^[47]^	2014	SAR_12	92,734 MMPs based upon retrosynthetic rules for 435 AC
41 ^[8]^	2014	SAR_13	20,073 and 25,297 MMP-based activity cliffs with K _i_ and IC _50_ values, respectively
42 ^[8]^	2014	SAR_14	4 activity measurement type-dependent sets of SAR transfer series with approximate or regular potency progression
43 ^[8]^	2014	SAR_15	169,889 and 240,322 transformation size-restricted MMPs based upon retrosynthetic rules with K _i_ and IC _50_ values, respectively

Data entries are organized according to scientific subject areas: structure-activity relationship (SAR) and structure-selectivity relationship (SSR) analysis, SAR visualization (SAR_VZ), virtual screening *via* similarity searching or machine learning (VS_ML), and programs (PROG). References in the Entry column provide the original publication introducing the program and/or data set. Program entries are described in more detail in Table 2 of our original data article ^1^. The new compound data sets 31–43 are discussed in the text. Programs and data sets reported herein have been separately deposited in ZENODO for access and download.

### Entry 31

50 compound activity classes (AC) are prioritized for the evaluation of scaffold hopping potential in ligand-based virtual screening ^38^. These AC contain the largest proportion of scaffold pairs with largest chemical inter-scaffold distances ^38^ that can be derived from current bioactive compounds and hence present challenging test cases for scaffold hopping analysis.

### Entry 32

596 SAR transfer series with regular potency progression (SAR-TS-RP) are extracted from 61 AC ^39^. Each SAR-TS-RP represents two compound series with different core structures and pairwise corresponding substitutions that yield comparable potency progression against a given target. These series provide a knowledge base for the analysis and prediction of SAR transfer events.

### Entry 33

Four sets of molecular scaffolds (with each scaffold representing more than ten compounds) are provided that are active against a single target (ST), multiple targets from the same family (SF), or multiple targets from different families (MF) ^40^. Data sets are separately assembled for different types of potency measurements ( *i.e*., K _i_ and IC _50_ values) and provide a resource of scaffolds representing compounds with varying degrees of target promiscuity.

### Entry 34

Two multi-target compound data sets consist of confirmed screening hits ^41^. Each set contains compounds with single-, dual-, and triple-target activity, or no activity. These data provide test cases for machine learning or other approaches to differentiate between compounds with overlapping yet distinct activity profiles.

### Entry 35

Four multi-target compound data sets are provided ^42^. Each set contains compounds tested in three different assays. Compounds are organized into eight different subsets according to their activity profiles, *i.e.*, single-, dual-, and triple-target activity, or no activity. In addition, three multi-mechanism compound sets are designed ^42^. In the latter case, compounds are organized into four subsets according to their mechanism-of-action. These data sets also represent test cases for machine learning to distinguish compounds with different activity profiles or mechanisms.

### Entry 36

2337 non-redundant compound series matrices (CSMs) are generated covering compounds active against a wide spectrum of targets ^43^. Each matrix contains at least two analogous matching molecular series (MMS) with structurally related yet distinct cores. A matrix consists of known active compounds and structurally related virtual compounds and hence provides suggestions for compound design.

### Entry 37

128 target-based data sets are assembled that consist of at least 100 compounds with precisely specified equilibrium constants (K _i_ values) below 1 µM for human targets ^44^. These high-confidence activity data sets provide a sound basis for SAR exploration.

### Entry 38

30,452 and 45,607 target-based MMS with K _i_ and IC _50_ values, respectively, are extracted from bioactive compounds ^45^.

### Entry 39

221 scaffolds are identified that only occur in approved drugs but are not found in currently available bioactive compounds ^46^. Accordingly, these scaffolds have been termed drug-unique scaffolds.

### Entry 40

92,734 MMPs are generated from 435 AC on a basis of retrosynthetic rules ^47^. These MMPs consider chemical reaction information and should be useful for practical medicinal chemistry applications.

### Entry 41

20,073 and 25,297 MMP-based activity cliffs ( *i.e.* pairs of structurally analogous compounds with an at least 100-fold difference in potency) are extracted from specifically active compounds based upon K _i_ and IC _50_ values, respectively ^8^. The MMP-based activity cliffs provide a large knowledge base for SAR analysis.

### Entry 42

157 and 513 MMP-based SAR transfer series with approximate potency progression plus 60 and 322 SAR transfer series with regular potency progression based upon K _i_ and IC _50_ values, respectively, are isolated from bioactive compounds. These transfer series are active against individual targets ^8^. Similar to MMP-based activity cliffs, SAR transfer series provide a resource for SAR analysis and compound design.

### Entry 43

169,889 and 240,322 transformation size-restricted MMPs based upon retrosynthetic rules with K _i_ and IC _50_ values, respectively, are systematically extracted from available AC ^8^. Different from the retrosynthetic rule-based MMPs presented above, applied transformation size-restrictions ensure that chemical changes distinguishing compounds in pairs are small.

## Summary

Herein we have provided an updated release of data sets and programs for chemoinformatics and medicinal chemistry that we make freely available. In total, 13 new data sets are introduced. Transferring all data entries in an organized form to the ZENODO platform makes them easily accessible. We hope that our current release might be of interest and helpful to many investigators in academia and the pharmaceutical industry.

## Data availability

ZENODO: Programs for chemoinformatics and computational medicinal chemistry, doi: 10.5281/zenodo.8451 ^48^.

ZENODO: Data sets for chemoinformatics and computational medicinal chemistry, doi: 10.5281/zenodo.8455 ^49^.
