# Supplementary material for: Follow up: Compound data sets and software tools for chemoinformatics and medicinal chemistry applications: update and data transfer
Source: F1000Res. 2014 Mar 11;3:69. [Version 1] doi: 10.12688/f1000research.3713.1 (PMC4264635; doi:10.12688/f1000research.3713.1)
Supplement: Peer review history

# Peer review: Referee response for version 1

**DOI:** 10.5256/f1000research.3979.r4077

**Published:** 2014-04-22

**Authors:** Patrick Walters

**Affiliations:** 1 Vertex Pharmaceuticals Incorporated, Cambridge, MA, USA

The ability to compare multiple computational methods across a series of consistent, high-quality datasets is critical to the progress of computational chemistry and cheminformatics. In the past, each paper published in the field seemed to present yet another new dataset. This dataset heterogeneity made it difficult, if not impossible, to objectively compare methods, and impeded the progress of the field. The availability of large repositories of carefully curated data is critical to the progress of the field. The datasets described in this paper will provide an invaluable resource for future studies. It is refreshing to see the emergence of platforms like ZENODO dedicated to hosting this data.

I have read this submission. I believe that I have an appropriate level of expertise to confirm that it is of an acceptable scientific standard.

# Peer review: Referee response for version 1

**DOI:** 10.5256/f1000research.3979.r4409

**Published:** 2014-04-17

**Authors:** Chris J. Swain

**Affiliations:** 1 Cambridge Med Chem Consulting, Cambridge, UK

Building and testing novel computer models requires access to suitable datasets. The authors have compiled a very useful set of interesting datasets and made them readily available in standard formats (SMILES and SDF). This allows others to both test existing algorithms and to develop new ones.

I have read this submission. I believe that I have an appropriate level of expertise to confirm that it is of an acceptable scientific standard.

# Peer review: Referee response for version 1

**DOI:** 10.5256/f1000research.3979.r4079

**Published:** 2014-03-13

**Authors:** Ajay Jain

**Affiliations:** 1 HDF Comprehensive Cancer Center, University of California San Francisco, San Francisco, CA, USA

Hu and Bajorath offer an update to their resource for computational chemistry. The curated data, and its engineered availability, will be of great interest, especially to methods developers. Even those researchers that are interested in exploring larger data sets that illuminate issues such as activity cliffs and small-molecule structural motifs will find the resource of interest.

I have read this submission. I believe that I have an appropriate level of expertise to confirm that it is of an acceptable scientific standard.
